# Hierarchical multimodal self-attention-based graph neural network for DTI prediction

**DOI:** 10.1093/bib/bbae293

**Published:** 2024-06-26

**Authors:** Jilong Bian, Hao Lu, Guanghui Dong, Guohua Wang

**Affiliations:** College of Computer and Control Engineering, Northeast Forestry University, No. 26 Hexing Road, Xiangfang District, Harbin, Heilongjiang 150040, China; College of Computer and Control Engineering, Northeast Forestry University, No. 26 Hexing Road, Xiangfang District, Harbin, Heilongjiang 150040, China; College of Computer and Control Engineering, Northeast Forestry University, No. 26 Hexing Road, Xiangfang District, Harbin, Heilongjiang 150040, China; College of Computer and Control Engineering, Northeast Forestry University, No. 26 Hexing Road, Xiangfang District, Harbin, Heilongjiang 150040, China

**Keywords:** DTI prediction, graph neural network, multimodal fusion, hierarchical multimodal self-attention

## Abstract

Drug–target interactions (DTIs) are a key part of drug development process and their accurate and efficient prediction can significantly boost development efficiency and reduce development time. Recent years have witnessed the rapid advancement of deep learning, resulting in an abundance of deep learning-based models for DTI prediction. However, most of these models used a single representation of drugs and proteins, making it difficult to comprehensively represent their characteristics. Multimodal data fusion can effectively compensate for the limitations of single-modal data. However, existing multimodal models for DTI prediction do not take into account both intra- and inter-modal interactions simultaneously, resulting in limited presentation capabilities of fused features and a reduction in DTI prediction accuracy. A hierarchical multimodal self-attention-based graph neural network for DTI prediction, called HMSA-DTI, is proposed to address multimodal feature fusion. Our proposed HMSA-DTI takes drug SMILES, drug molecular graphs, protein sequences and protein 2-mer sequences as inputs, and utilizes a hierarchical multimodal self-attention mechanism to achieve deep fusion of multimodal features of drugs and proteins, enabling the capture of intra- and inter-modal interactions between drugs and proteins. It is demonstrated that our proposed HMSA-DTI has significant advantages over other baseline methods on multiple evaluation metrics across five benchmark datasets.

## Introduction

Drug–target interaction (DTI) prediction is a critical part of the drug development process, but traditional screening experiments require a substantial investment of human and material resources, resulting in high drug development costs [1]. With the increasing number of compound libraries and target libraries, computational methods have become an effective tool for improving drug development efficiency and reducing research costs [2]. In general, there are two classes of methods for predicting DTIs: traditional machine learning methods and deep learning methods. Traditional machine learning methods utilize various descriptors to encode drugs and proteins, which are taken as inputs to random forest [3–5], support vector machine [6–8] and logistic regression [9–11] for predicting DTIs. With the rapid increase in bioactivity data, data distribution has become more complex. Due to its ability to automatically extract features and learn complex nonlinear relationships, deep learning is well suited to handling complex data distributions. Consequently, it is increasingly employed in DTI prediction, showcasing impressive performance.

However, deep learning methods take drugs and proteins as inputs and automatically extract features of drugs and targets through network models to DTIs [12]. Recent developments in deep learning have advanced the field of DTI prediction, yielding a wide variety of methods for DTI prediction. Öztürk *et al*. [13] presented DeepDTA, in which one convolutional neural network (CNN) module for drug SMILES and the other CNN module for protein sequences are employed to extract their features for drug–target affinity prediction. Thereafter, Lee *et al*. [14] proposed DeepConvDTI, which puts protein sequences and drug fingerprints into multi-scale 1D convolutional modules and fully connected layers, respectively, for the extraction of their features. Abbasi *et al*. [15] presented DeepCDA, which learns compound and protein features by combining CNN and long short-term memory, and employs two-sided attention to compute their interactions. With Transformer [16] achieving extraordinary success in machine translation tasks, a number of transformer-based DTI prediction models have been developed. Chen *et al*. [17] developed TransformerCPI, in which gated convolutional network and graph convolutional network (GCN) are employed to extract protein and drug features, respectively. Then a modified transformer is utilized to calculate interaction features for DTI prediction.

Although sequence-based DTI prediction has made remarkable progress, it has limited capabilities to capture a molecule’s chemical structure, consequently adversely affecting DTI prediction. A graph-based representation can describe the topological structure of a molecule, beneficial to DTI prediction. Consequently, graph neural networks (GNNs) have been widely applied to DTI prediction, delivering superior predictive performance. Tsubaki *et al*. [18] utilized GNN and CNN to compute drug and protein features, respectively. They were concatenated and then fed into the classification network for prediction. Li *et al*. [19] utilized drug molecular graphs and 2D distance maps to represent drugs and proteins. The 2D distance maps are taken as inputs to ResNet to compute protein features. The protein features and drug molecular graphs are taken as inputs to the Interformer with interaction attention to compute interaction features. Wang *et al*. [20] proposed CSConv2d by improving DEEPScreen [21]. The CSConv2d uses 2D images to represent compounds, and uses channel attention and spatial attention to improve its prediction performance. Torng *et al*. [22] proposed a GNN-based method for DTI prediction. This method firstly pretrains a protein pocket graph AutoEncoder and utilizes the encoder module to extract protein features. It then employs GCN to extract drug features, achieving impressive results in DTI prediction. Bai *et al*. [23] learned local interaction representations between drugs and targets through a bilinear attention network and took them as inputs to fully connected layers for DTI prediction, in which conditional domain adversarial learning was applied for better generalization.

Each modality has its own distinctive information different from other modalities. The fusion of these modalities can provide more comprehensive and richer information, which helps the model better understand and depict the features and relationships within the data, further enhancing the model’s learning and generalization capabilities [24]. Therefore, multimodal data are also widely applied to DTI prediction to improve prediction accuracy. Wu *et al*. [25] combined SMILES with Morgan fingerprints to represent drugs, and protein sequences with k-mer sequences to represent proteins. Then, CNN was used to extract drug and protein features as hidden states of nodes, and virtual nodes were added to bridge drugs and proteins to form an association graph for DTI prediction. Wang *et al*. [26] proposed MCPI, which extracts from protein–protein interaction network, compound–compound interaction network, Morgan fingerprint, drug–molecule distance matrix and protein sequence the drug and protein features that are fused into drug–protein features, followed by fully connected layers for DTI prediction. Hua *et al*. [27] proposed CPInformer for DTI prediction. The graph features extracted by GCN and the FCFP features obtained by fully connected layers from FCFPs are fused into compound features. Additionally, the multi-scale protein features are extracted by multi-scale convolutions from protein sequences. Finally, these two features are fused by the ProbSparse attention mechanism [28] and sequentially fed into convolutional layers and fully connected layers for predicting DTIs.

Although multimodal-based DTI prediction has made significant progress, they still face challenges in effectively fusing multimodal data. Most DTI prediction models often ignored the intra-modal interactions, when modeling the inter-modal interactions, thus failing to model inter- and intra-modal interactions simultaneously. In this study, to overcome the abovementioned drawbacks, we present a hierarchical multimodal self-attention-based GNN for DTI prediction, referred to as HMSA-DTI. Each representation of drugs and proteins has its own distinctive information. For different representations of drugs and proteins, different feature extraction methods are employed to compute their features, which are then combined to improve their feature quality. Since graph-level features have an adverse effect on the multimodal feature fusion, the readout phase is removed from GNN and node-level features are kept for the multimodal feature fusion. In order to fuse the multimodal features, we propose a hierarchical multimodal self-attention to extract more fine-grained interaction features of multiple modalities of drugs and proteins. In ablation experiments, we demonstrate the benefits of our proposed hierarchical multimodal self-attention in multimodal feature fusion. Furthermore, experimental results show that our proposed HMSA-DTI outperforms some state-of-the-art methods for DTI prediction.

## Methods

### Overview of HMSA-DTI

**Figure 1:**
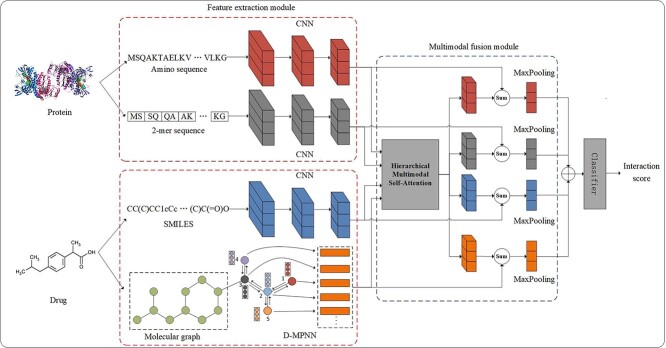
The overall architecture of HMSA-DTI. After mapping each of the four inputs to the embedding space, HMSA-DTI uses CNN and D-MPNN to extract features, fuses the multimodal features with a hierarchical multimodal self-attention mechanism to capture DTIs and finally the classifier outputs the predicted scores.

We present a hierarchical multimodal self-attention GNN (short for HMSA-DTI), as illustrated in Fig. 1. The overall framework of HMSA-DTI is composed of four components: protein feature extraction component, drug feature extraction component, multimodal feature fusion component and classifier component. In the protein feature extraction component, protein features are extracted using a CNN block with protein sequences and 2-mer sequences as input. In the drug feature extraction component, drug SMILES and molecular graphs are utilized as inputs. CNN and GNN are used to extract drug features. To capture and exploit the complex interactions between proteins and drugs to enhance their feature representation capability, we present a hierarchical multimodal self-attention mechanism. Using this hierarchical multimodal self-attention mechanism, the four features are fused and then concatenated as inputs to the classifier component for identification of DTIs.

### Feature extraction for proteins

In the protein extraction module, we extract both global and local structural features of proteins to better characterize their properties. The global structural features of proteins are extracted from their sequences by a convolution block, while the local structural features are extracted from their k-mer sequences [29].

#### Feature extraction from protein sequences

In the same manner as Rao *et al*. [30], a protein sequence is encoded into a numerical vector and zero padding is applied to obtain a numerical vector of equal length $x_{_{token}}$. Then, an embedding layer is utilized to map the numerical vectors to the embedding space, resulting in the generation of the embedding matrix. This matrix is subsequently fed into a CNN block to extract features. 


(1) \begin{align*} X_{_{seq}}=CNN_{_{seq}}(Emb(x_{_{token}})),\end{align*}

where $X_{_{seq}}\in \mathbb{R}^{L_{seq}\times d_{protein}}$, and $L_{_{seq}}$ and $d_{_{protein}}$ represent the spatial dimension and embedding dimension of the protein sequence feature, respectively. $Emb$ is an Embedding layer.

#### Feature extraction from 2-mer protein sequences

The sliding window algorithm is used to obtain k-mer protein sequences. Specifically, a window of length k is created and slides from left to right, shifting one amino acid at a time. If the length of the protein sequence is N, we would obtain N-k+1 k-mer. In this paper, k is set to 2, ending up with a 2-mer protein sequence $x_{_{mer}}$. After that, 2-mer sequences are encoded into numerical vectors using HyperAttentionDTI’s encoding method [31]. 


(2) \begin{align*} X_{_{mer}}=CNN_{_{mer}}(Enc(x_{_{mer}})),\end{align*}

where $X_{_{mer}}\in \mathbb{R}^{L_{_{mer}}\times d_{protein}}$, and $L_{_{mer}}$ is the spatial dimension of the 2-mer sequence feature. $Enc$ is the numerical vector encoder in HyperAttentionDTI [31]. This 2-mer sequence feature serves as its local feature representation.

### Feature extraction for drugs

Drugs are typically represented as SMILES or molecular graphs. SMILES is widely employed in computational chemistry, chemoinformatics and bioinformatics due to its compact storage and easy recognition. Molecular graphs can capture the local and global context of atoms and bonds within molecules and powerful GNNs can extract features from them. In this paper, both SMILES and molecular graphs are used as drug representations to enhance feature robustness.

#### Features extraction from SMILES

When SMILES for a drug molecule is taken as input to a neural network, it needs to be converted into a digital vector. In this paper, the characters in SMILES are converted to integers from 1 to 64 to form a digital vector, and then it is padded with 0s to produce a vector of fixed length $x_{_{smiles}}$, which is subsequently mapped to an embedding space. Finally, a drug SMILES feature $X_{_{smiles}}$ is obtained using a CNN block $CNN_{_{smiles}}$: 


(3) \begin{align*} X_{_{smiles}}=CNN_{_{smiles}}(Emb(x_{_{smiles}})),\end{align*}

where $X_{_{smiles}}\in \mathbb{R}^{L_{s}\times d_{_{drug}}}$, $L_{s}$ is the spatial dimension and $d_{_{drug}}$ is the embedding dimension.

#### Feature extraction from molecular graph

A drug molecule graph $G = (A,B)$ is composed of a set of atoms acting as its nodes, represented by $A$, and a set of chemical bonds serving as its edges, denoted by $B$. In this representation, nodes correspond to atoms in the drug molecule, while edges represent the chemical bonds between these atoms. In this study, the initial feature $x_{v}$ of node $v$ consists of eight properties: atomic number, degree (number of chemical bonds), formal charge, chirality, number of hydrogen atoms attached to the atom, hybridization, aromaticity and atomic mass. The initial feature of edge $e_{vw}$ includes bond type, bond position, conjugation and stereochemistry. Then a directed message passing network (D-MPNN) [32] is used to extract molecular graph features, which is composed of a message passing stage and readout stage. In contrast to traditional MPNN, D-MPNN not only uses the features of the nodes but also the directionality of edges in the process of message passing updates. For any node $v$ and the edge $B_{vw}$ associated with it, $h^{t}_{v}$ and $B^{t}_{vw}$ denote their hidden states at the $t$-th layer, respectively. The initial feature $x_{v}$ of node $v$ is used as the initial hidden state $h_{v}^{0}$, while the hidden state $h_{vw}^{0}$ of edge $B_{vw}$ is given by 


(4) \begin{align*} h_{vw}^{0}=\alpha (W_{b}(concat(x_{v},e_{vw}))),\end{align*}

where $W_{b}$ is a parameter matrix, $\alpha $ denotes the activation function $ReLu$ and $concat$ represents a concatenation layer. The message passing stage first aggregates the features $x_{v}$ of node $v$, the feature $x_{k}$ of its neighbor node $k$ and the hidden state $h_{kv}^{t}$ of edge $B_{kv}$ to obtain the message $m_{vw}^{t+1}$ of edge $B_{vw}$ at $t+1$-th layer, which is given by 


(5) \begin{align*} m_{vw}^{t+1}=\sum_{k\in \{N(v)\backslash{w}\}}M(x_{v},x_{k},h_{kv}^{t}),\end{align*}

where $M$ denotes an average function and $N(v)$ represents the set of neighbor nodes for node $v$. Then, the message $m_{vw}^{t+1}$ and the hidden state $h_{vw}^{t}$ are utilized to calculate the hidden state $h_{vw}^{t+1}$ of edge $B_{vw}$ at $t+1$-th layer: 


(6) \begin{align*} h_{vw}^{t+1}=f(h_{vw}^{t}, m_{vw}^{t+1}),\end{align*}

where $f$ is a fully connected layer. After $T$ iterations, we obtain the final hidden states of all edges. Then, the hidden states of edges connected to node $v$ are aggregated to compute the message $m_{v}$ of node $v$, which is concatenated with its initial feature $x_{v}$ to compute its final hidden state $h_{v}$: 


(7) \begin{align*}& \begin{aligned} m_{v}&=\sum_{k\in{N(v)}}h_{kv}^{T}\\ h_{v}&=\alpha (W_{\alpha}(concat(x_{v},m_{v}))) \end{aligned},\end{align*}

where $W_{\alpha }$ is a parameter matrix.

In general, at the readout stage, average pooling or max pooling is used to pool the features from nodes to produce a single vector, that is, a graph-level feature. Since drug properties are closely related to their local structures, the pooling operation at the readout stage causes the loss of node-level features, resulting in a reduction in the accuracy of DTI prediction [33]. Previous studies usually used graph-level features of drug molecular graphs to fuse with other modal features. However, multimodal fusion using graph-level features cannot capture both inter- and intra-modal interactions simultaneously. Therefore, in this paper, node-level features $X_{_{graph}}\in{\mathbb{R}^{L_{g}\times d_{drug}}}$ rather than graph-level features are used for DTI prediction, where $L_{g}$ is the atomic number of a drug molecule, and $d_{_{drug}}$ is its embedding dimension. Finally, our proposed hierarchical multimodal self-attention is used to fuse multimodal features of drugs and proteins to improve their feature representation ability.

### Hierarchical multimodal self-attention

**Figure 2:**
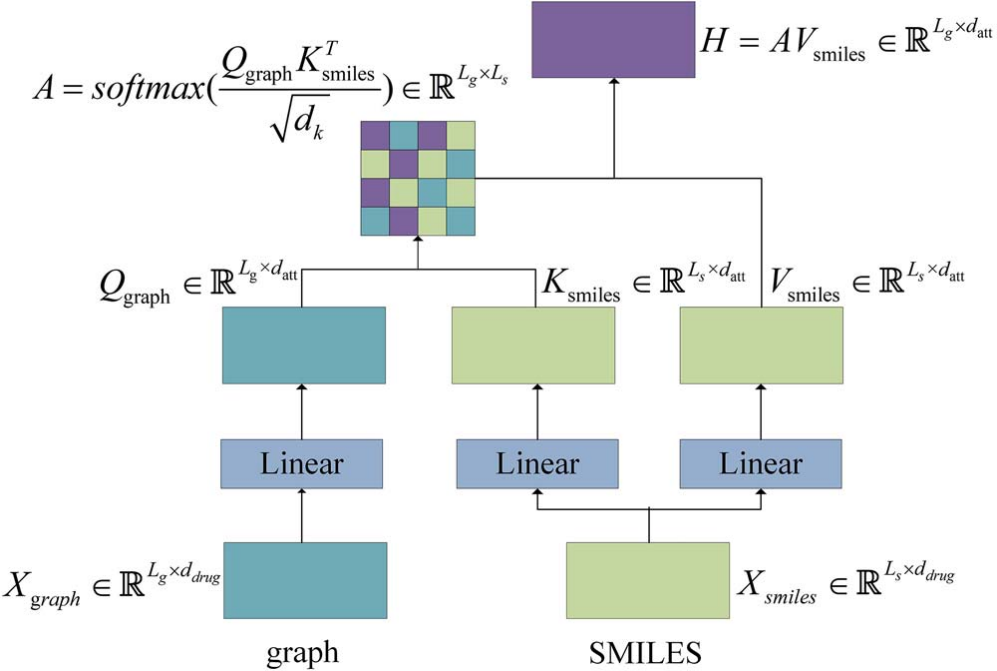
The diagram for cross-attention.


**Figure 3:**
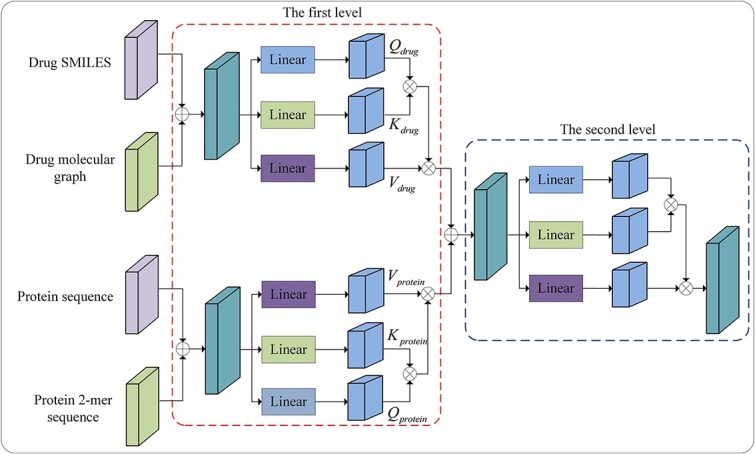
Hierarchical multimodal self-attention mechanism. The red dashed box and blue dashed box represent the first-level and second-level attention modules, respectively. $\oplus $ denotes the concatenation operation and $\otimes $ denotes the matrix multiplication.


Multimodal learning is able to enhance feature representation by fusing features from multiple data sources to improve prediction performance [34]. In the DTI prediction task, the accuracy of the prediction can be improved by combining features from drug SMILES, drug molecular graphs, protein sequences and protein 2-mer sequences. The simplest multimodal feature fusion is to concatenate features from different modes without considering inter-modal relationships. As a result, the fused features lose semantic information and inter-modal interaction information. Many research works used cross-attention to fuse multi-modal features. As far as drug representations is concerned, SMILES describes the composition of drug molecules in a linear manner, while molecular graph describes the topological relationships between atoms using a graph structure. The fusion of these two representations can enhance the model’s capacity to capture drug molecules and allows extracting more robust drug features. The process for the fusion of drug features using cross-attention is shown in Fig. 2. A linear transformation is applied to the drug molecular graph feature $X_{_{graph}}\in{\mathbb{R}^{L_{g}\times d_{drug}}}$ to compute Query matrix $Q_{_{graph}}\in{\mathbb{R}^{L_{g}\times d_{att}}}$, and to the drug SMILES feature $X_{_{smiles}}\in{\mathbb{R}^{L_{s}\times d_{drug}}}$ to compute Key matrix $K_{_{smiles}}\in{\mathbb{R}^{L_{s}\times d_{att}}}$ and Value matrix $V_{_{smiles}}\in{\mathbb{R}^{L_{s}\times d_{att}}}$:

(8) \begin{align*}& \begin{aligned} Q_{_{graph}}&=Linear(X_{_{graph}})=X_{_{graph}}W_{_{Q}}+b_{_{Q}} \\ K_{_{smiles}}&=Linear(X_{_{smiles}})=X_{_{smiles}}W_{_{K}}+b_{_{K}} \\ V_{_{smiles}}&=Linear(X_{_{smiles}})=X_{_{smiles}}W_{_{V}}+b_{_{V}} \end{aligned},\end{align*}

where $W_{_{Q}}$, $W_{_{K}}$ and $W_{_{V}}$ are parameter matrices and $b_{_{Q}}$, $b_{_{K}}$ and $b_{_{V}}$ are parameter vectors. It is followed by that the Query matrix $Q_{_{graph}}\in{\mathbb{R}^{L_{g}\times d_{att}}}$ and Key matrix $K_{_{smiles}}\in{\mathbb{R}^{L_{s}\times d_{att}}}$ are used to compute the attention matrix $A\in{\mathbb{R}^{L_{g}\times L_{s}}}$: 


(9) \begin{align*} A=softmax\left(\frac{Q_{_{graph}}K_{_{smiles}}^{T}}{\sqrt{d_{k}}}\right),\end{align*}

where $d_{k}=d_{att}$. Then, the attention matrix $A$ and value matrix $V_{_{smiles}}$ are used to fuse the SMILES features and the graph features: 


(10) \begin{align*} H=AV_{_{smiles}}= \begin{bmatrix} a_{_{11}}v_{_{1}}+a_{_{12}}v_{_{2}}+...+a_{_{1L_{s}}}v_{_{L_{s}}} \\ a_{_{21}}v_{_{1}}+a_{_{22}}v_{_{2}}+...+a_{_{2L_{s}}}v_{_{L_{s}}} \\ ...\\ a_{_{L_{g}1}}v_{_{1}}+a_{_{L_{g}2}}v_{_{2}}+...+a_{_{L_{g}L_{s}}}v_{_{L_{s}}} \\ \end{bmatrix},\end{align*}

where $a_{ij}$ is an element of the attention matrix $A$, and $v_{i}$ represents the $i$-th row of the value matrix $V_{_{smiles}}$. Eq. (10) shows that the fusion feature $H$ generated by cross-attention is just a weighted combination of the row vectors in the value matrix $V_{_{smiles}}$ which is a linear transform of the drug SMILES feature, resulting in being short of the drug molecular graph feature. This fusion method does not take full advantage of multimodal features. In this study, we present a hierarchical multimodal self-attention mechanism to fuse multimodal features from drugs and proteins. This mechanism can capture and exploit the complex interactions between proteins and drugs, taking into account both intra- and inter-modal interactions. As illustrated in Fig. 3, this attention mechanism includes two levels. At the first level, the drug SMILES feature and molecular graph feature are fused to compute the first-level drug fusion feature $H_{1}^{^{drug}}$. Similarly, the protein sequence feature and protein 2-mer feature are also fused to obtain the first-level protein fusion feature $H_{1}^{^{protein}}$. We first concatenate the smiles feature $X_{_{smiles}}$ with the graph feature $X_{_{graph}}$ to obtain the combined drugs feature $H_{_{drug}}^{^{concat}}$. Next, three linear transformations are applied to the combined drug feature $H_{_{drug}}^{^{concat}}$ to compute Query matrix $Q_{_{drug}}$, Key matrix $K_{_{drug}}$ and Value matrix $V_{_{drug}}$:

(11) \begin{align*}& \begin{aligned} Q_{_{drug}}&=H_{_{drug}}^{^{concat}}W_{_{Q}}+b_{_{Q}}=\begin{bmatrix}X_{_{smiles}}\\X_{_{graph}}\end{bmatrix}W_{_{Q}}+b_{_{Q}}=\begin{bmatrix}Q_{_{smiles}}\\Q_{_{graph}}\end{bmatrix}\\ K_{_{drug}}&=H_{_{drug}}^{^{concat}}W_{_{K}}+b_{_{K}}=\begin{bmatrix}X_{_{smiles}}\\X_{_{graph}}\end{bmatrix}W_{_{K}}+b_{_{K}}=\begin{bmatrix}K_{_{smiles}}\\K_{_{graph}}\end{bmatrix}\\ V_{_{drug}}&=H_{_{drug}}^{^{concat}}W_{_{V}}+b_{_{V}}=\begin{bmatrix}X_{_{smiles}}\\X_{_{graph}}\end{bmatrix}W_{_{V}}+b_{_{V}}=\begin{bmatrix}V_{_{smiles}}\\V_{_{graph}}\end{bmatrix} \end{aligned}\end{align*}

As shown in Eq. (11), $Q_{_{drug}}$, $K_{_{drug}}$ and $V_{_{drug}}$ include both SMILES features and molecular graph features of the drug. Then Query matrix $Q_{_{drug}}$ and Key matrix $K_{_{drug}}$ are used to compute the attention matrix $A_{_{drug}}$: 


(12) \begin{align*}\begin{aligned} A_{_{drug}}&=softmax\left(\frac{Q_{_{drug}}K_{_{drug}}^{T}}{\sqrt{d_{k}}} \right)\\ &=softmax\left(\frac{\begin{bmatrix} Q_{_{smiles}}K_{_{smiles}}^{T} & Q_{_{smiles}}K_{_{graph}}^{T}\\ Q_{_{graph}}K_{_{smiles}}^{T} & Q_{_{graph}}K_{_{graph}}^{T} \end{bmatrix}}{\sqrt{d_{k}}}\right) \end{aligned}\end{align*}

Eq. (12) shows that the drug attention matrix $A_{_{drug}}$ includes correlations between SMILES and SMILES, between SMILES and molecular graphs, between molecular graphs and SMILES and between molecular graphs and molecular graphs. Then the drug fusion feature $H_{1}^{drug}$ is given by 


(13) \begin{align*}& \begin{aligned} H_{1}^{_{drug}}&=A_{_{drug}}V_{_{drug}}\\ &=\begin{bmatrix} Q_{_{smiles}}K_{_{smiles}}^{T} & Q_{_{smiles}}K_{_{graph}}^{T}\\ Q_{_{graph}}K_{_{smiles}}^{T} & Q_{_{graph}}K_{_{graph}}^{T} \end{bmatrix}\times \begin{bmatrix} V_{_{smiles}}\\ V_{_{graph}} \end{bmatrix}\\ &=\begin{bmatrix} Q_{_{smiles}}K_{_{smiles}}^{T}V_{_{smiles}} + Q_{_{smiles}}K_{_{graph}}^{T}V_{_{graph}}\\ Q_{_{graph}}K_{_{smiles}}^{T}V_{_{smiles}} + Q_{_{graph}}K_{_{graph}}^{T}V_{_{graph}} \end{bmatrix}, \end{aligned}\end{align*}

where the denominator $\sqrt{d_{k}}$ and the $softmax$ function are omitted to simplify the expression. It can be seen in Eq. (13) that the first-level drug fusion feature $H_{1}^{_{drug}}$ includes SMILES features as well as molecular graph features. Similarly, the same feature fusion process is applied to protein sequence features and 2-mer sequence features to compute the first-level protein fusion feature $H_{1}^{_{protein}}$. At the second level, $H_{1}^{_{protein}}$ and $H_{1}^{_{drug}}$ are processed in a manner similar to the first level in order to compute the final drug-target feature $H$. The drug–target feature $H$ is split into the drug SMILES feature $D_{_{smiles}}$, drug graph feature $D_{_{graph}}$, protein sequence feature $D_{_{seq}}$ and protein 2-mer sequence feature $D_{_{mer}}$, which are added to their initial features, that is, inputs to hierarchical multimodal self-attention. Lastly, a pooling layer is employed to compute the drug SMILES feature vector $Z_{_{smiles}}$, the drug molecular graph feature vector $Z_{_{graph}}$, the protein sequence feature vector $Z_{_{seq}}$ and the protein 2-mer sequence feature vector $Z_{_{mer}}$ as the outputs of the multimodal feature fusion module:


(14) \begin{align*}& \begin{aligned} Z_{_{smiles}}&=maxpooling(D_{_{smiles}}+X_{_{smiles}})\\ Z_{_{graph}}&=maxpooling(D_{_{graph}}+X_{_{graph}})\\ Z_{_{seq}}&=maxpooling(D_{_{seq}}+X_{_{seq}})\\ Z_{_{mer}}&=maxpooling(D_{_{mer}}+X_{_{mer}}) \end{aligned}\end{align*}

### Classifier and loss function

In the classifier component, we employed four fully connected layers, each followed by a dropout layer and a LeakyReLU activation function. In addition, binary cross-entropy loss is used: 


(15) \begin{align*} L=-\sum_{i=1}^{n}{t_{i}log(p_{i})+(1-t_{i})log(1-p_{i})},\end{align*}

where $t_{i}$ is the sample label with positive sample being 1 and negative sample being 0, and $p_{i}$ is the predicted probability.

## Results

### Benchmark datasets

Our HMSA-DTI model and four baseline models were assessed on five widely used benchmark datasets: DrugBank, Human, C.elegans, BioSNAP and Davis. The Human and C.elegans are balanced datasets, which were created by Liu [35]. The DrugBank is also a balanced dataset, which was created by HyperAttentionDTI [31]. The BioSNAP is sourced from the Stanford Biomedical Network Dataset [36], and we constructed an imbalanced dataset with a positive-to-negative sample ratio of 1:10 based on this dataset. The Davis [37] is an unbalanced dataset, including 68 drugs and 379 proteins. In Table 1, a detailed description of these datasets is provided.

**Table 1:** The detailed description of benchmark datasets

Dataset	Protein	Drug	Negative	Positive
Human	852	1052	3387	3364
C.elegans	2504	1434	3893	3893
DrugBank	4254	6645	17 511	17 511
Davis	379	68	18 452	7320
BioSNAP	2181	4509	138 340	13 834

### Experimental analysis

In this paper, we used AUC, ACC(Accuracy), Precision and Recall to evaluate the model performance on the balanced dataset and AUC and AUPR to evaluate the model performance on the unbalanced dataset. We compared our proposed HMSA-DTI model with four state-of-the-art methods: HyperAttentionDTI [31], GIFDTI [38], CoaDTI [39], MHSADTI [40]. For fair comparison, all models were evaluated using 10-fold cross validation on a Linux server equipped with a GeForce RTX 3090 GPU. The hyperparameters in comparison models were set in accordance with the corresponding literature. During the experimental process, 10% of the data were selected as the test dataset, while the remaining data were divided into training and validation datasets. The test dataset is fixed across experiments and the data from the test dataset do not appear in the training and validation datasets of any cross-validation experiment. During training, the number of epochs and the batch size were set to 150 and 50, respectively, with the best-performing model on the validation dataset saved for testing on the test dataset. The mean of evaluation metrics for the 10-fold cross-validation results was reported.

#### Performance comparison on the DrugBank dataset

**Figure 4:**
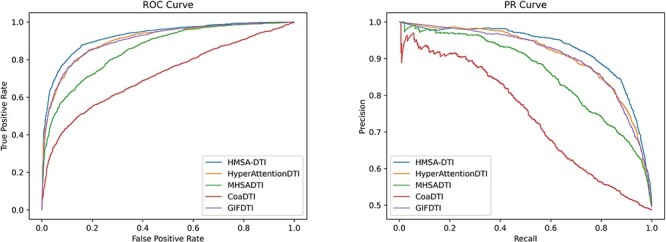
ROC curve and PR curve of the HMSA-DTI model and baseline models on DrugBank dataset.

This section presents a comparative analysis of the proposed HMSA-DTI model and four baseline models using the DrugBank dataset. The results of the experiment, as presented in Table 2, clearly demonstrate the outstanding performance of our HMSA-DTI model with respect to four evaluation metrics: AUC, precision, recall and ACC. Compared with the top-performing baseline GIFDTI, the HMSA-DTI achieved a large improvement of 1.38% in AUC, 2.14% in precision, 0.41% in recall and 1.84% in ACC. There are two main factors that contribute to this significant improvement. Firstly, our HMSA-DTI model takes off the readout phase in D-MPNN, allowing node-level features to be retained rather than graph-level features. This is beneficial to a thorough fusion of multimodal data from both drugs and proteins using our hierarchical multimodal self-attention. Secondly, the hierarchical multimodal self-attention based feature fusion approach allows the simultaneous extraction of inter- and intra-modal interactions, resulting in improved feature representation capabilities. To better illustrate the differences in model performance, we provide the PR and ROC curves of the evaluated models on the DrugBank dataset, as displayed in Fig. 4. It is demonstrated that the HMSA-DTI model consistently outperforms the baseline models in prediction performance, showing its superiority and effectiveness in DTI prediction.

**Table 2:** The performance comparison between the HMSA-DTI model and the baseline models on the DrugBank dataset

Model	AUC	ACC	Precision	Recall
HMSA-DTI	**0.9218**	**0.8474**	**0.8371**	**0.8598**
CoaDTI	0.7541	0.6790	0.7215	0.5781
MHSADTI	0.8591	0.7743	0.7551	0.8076
HyperAttentionDTI	0.9051	0.8245	0.8134	0.8477
GIFDTI	0.9080	0.8290	0.8157	0.8557

#### Performance comparison on the Human dataset

This section provides a performance comparison of HMSA-DTI model and four baseline models on the Human dataset. As illustrated in Table 3, our HMSA-DTI model shows superior performance on four metrics: AUC, precision, ACC and recall. Against the best-performing baseline model, HyperAttentionDTI, HMSA-DTI achieves improvements of 1.32% in AUC, 0.99% in precision and 1.26% in ACC. Furthermore, HMSA-DTI shows an improvement of 1.33% over the CoaDTI model, which ranks first in terms of a recall metric among all baseline models. It is shown that our HMSA-DTI model has excellent predictive performance on a Human dataset.

**Table 3:** The performance comparison between the HMSA-DTI model and the baseline models on the Human dataset

Model	AUC	ACC	Precision	Recall
HMSA-DTI	**0.9937**	**0.9589**	**0.9591**	**0.9528**
CoaDTI	0.9700	0.9154	0.9061	0.9395
MHSADTI	0.9693	0.9117	0.8992	0.9311
HyperAttentionDTI	0.9805	0.9463	0.9492	0.9375
GIFDTI	0.9715	0.9140	0.9022	0.9197

#### Performance comparison on the C.elegans dataset

A comparison between HMSA-DTI model and four baseline models was made on the C.elegans dataset in this section. As shown in Table 4, compared with the best-performing baseline model HyperAttentionDTI on this dataset, HMSA-DTI achieved an improvement of 0.18%, 0.19% and 0.46% in terms of AUC, ACC and recall, respectively. Experimental results on the three aforementioned benchmark datasets demonstrate that our HMSA-DTI model is highly competitive and effective.

**Table 4:** The performance comparison between the HMSA-DTI model and the baseline models on the C.elegans dataset

Model	AUC	ACC	Precision	Recall
HMSA-DTI	**0.9950**	**0.9667**	0.9613	**0.9746**
CoaDTI	0.9710	0.9278	0.9373	0.9228
MHSADTI	0.9850	0.9432	0.9344	0.9614
HyperAttentionDTI	0.9932	0.9648	**0.9660**	0.9700
GIFDTI	0.9848	0.9461	0.9343	0.9526

#### Performance comparison on the Davis dataset

**Figure 5:**
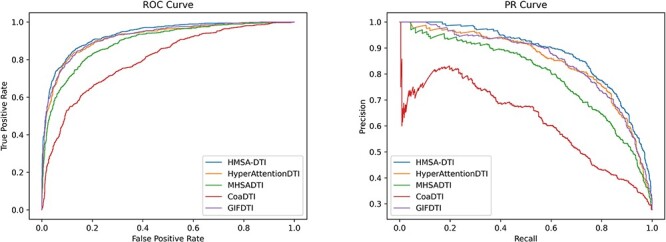
ROC curve and PR curve of the HMSA-DTI model and baseline models on Davis dataset.

In this section, we compared the performance of the HMSA-DTI model with four baseline models on the Davis dataset. Since the Davis dataset is an unbalanced dataset, we used AUC and AUPR to evaluate these models. As shown in Table 5, compared with the best-performing baseline model HyperAttentionDTI, our HMSA-DTI achieved an improvement of 0.2% and 1.12% in terms of AUC and AUPR, respectively. To better illustrate the differences in model performance, the PR and ROC curves on the Davis dataset are displayed in Fig. 5.

**Table 5:** The performance comparison between the HMSA-DTI model and the baseline models on the Davis dataset

Model	AUC	AUPR
HMSA-DTI	**0.9327**	**0.8699**
CoaDTI	0.8100	0.6468
MHSADTI	0.8876	0.7537
HyperAttentionDTI	0.9303	0.8587
GIFDTI	0.9225	0.8498

#### Performance comparison on an independent dataset

Although our HMSA-DTI model outperformed the baseline models on the benchmark Datasets, to verify the generalization ability of the models, we trained our model on the DrugBank dataset and tested it on the BioSNAP dataset. During training, 80% of the DrugBank dataset was used as the training dataset, while the remaining 20% of the data were used as the validation dataset. All data from the BioSNAP dataset served as the test dataset. The experimental results in Table 6 show that compared with all baselines, HMSA-DTI outperformed all baselines by at least 3% on both AUC and AUPR metrics.

**Table 6:** The performance comparison between the HMSA-DTI model and the baseline models on the independent dataset

Model	AUC	AUPR
HMSA-DTI	**0.8877**	**0.5812**
CoaDTI	0.6795	0.2034
MHSADTI	0.8132	0.4928
HyperAttentionDTI	0.8495	0.4938
GIFDTI	0.8483	0.4441

### Ablation experiments

A set of ablation experiments was carried out on the DrugBank dataset to test the effectiveness of the various modules within our model, and the results are shown in Table 7. We first replaced the hierarchical multimodal self-attention based feature fusion method in the HMSA-DTI with the concatenation-based feature fusion method to obtain the HMSA-DTI-Concat model. According to our results, we found that the HMSA-DTI, which includes hierarchical multimodal self-attention, outperforms the HMSA-DTI-Concat model on all four evaluation metrics. Our HMSA-DTI model outperforms the HMSA-DTI-Concat model by 0.61% in AUC, 0.72% in ACC, 0.76% in precision and 0.64% in recall metrics, respectively. It is shown that our hierarchical multimodal self-attention mechanism is beneficial for fusing multimodal features and can improve the feature representation ability of drugs and proteins. In addition, we used two attention variants Non-Local [41] and Cross-Attention [42] to construct HMSA-DTI-NL and HMSA-DTI-CA, respectively. It is found that the HMSA-DTI model improved AUC by 0.68%, ACC by 0.5%, precision by 1.18% and recall by 0.46% compared with the HMSADTI-NL model. In comparison with the HMSA-CA model, the HMSA-DTI model demonstrated significant strengths in several metrics with 2.71% higher AUC, 4.29% more ACC, 4.35% better precision and 3.15% higher recall. To validate the impact of the combination of different representations of drug molecules on prediction performance, we replaced the combination of SMILES and molecular graph with the combination of ECFP and molecular graph to obtain the HMSA-DTI-ECFP model. The results indicate that our HMSA-DTI model using the combination of SMILES and molecular graph outperforms the HMSA-DTI-ECFP model by 0.82% in AUC, 1.36% in ACC, 1.92% in precision and 0.37% in recall, respectively. To validate the contribution of the combination of global and local features of proteins to prediction performance, we removed the 2-mer sequences of proteins from the HMSA-DTI model to obtain the HMSA-DTI-Without2mer model. The results demonstrate that the combination of protein sequences and 2-mer sequences in the HMSA-DTI model leads to improvements of 0.71% in AUC, 0.71% in ACC, 1.3% in precision and 0.75% in recall, respectively. It is shown that multi-source data fusion based on hierarchical multimodal self-attention can effectively enhance the representation capability of fused features.

**Table 7:** Ablation experiments on DrugBank dataset

Model	AUC	ACC	Precision	Recall
HMSA-DTI	**0.9218**	**0.8474**	**0.8371**	**0.8598**
HMSA-DTI-Concat	0.9157	0.8402	0.8295	0.8534
HMSA-DTI-NL	0.9150	0.8424	0.8253	0.8552
HMSA-DTI-CA	0.8947	0.8045	0.7936	0.8283
HMSA-DTI-ECFP	0.9136	0.8338	0.8179	0.8561
HMSA-DTI-Without2mer	0.9147	0.8403	0.8241	0.8523

### Parameter sensitivity analysis

#### K-value analysis

**Figure 6:**
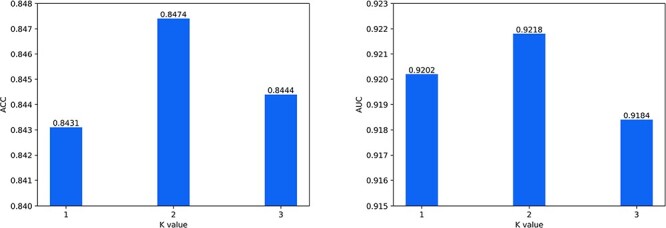
The impact of K value on ACC and AUC.

When proteins are represented by k-mer sequences, the choice of K-value will impact model performance. In general, a smaller value of K makes the model focus more on the local structure of proteins, whereas a larger value of K makes the model puts more emphasis on the global structure of proteins. When K=1, 2, 3, the corresponding vocabulary sizes are 22, $22^{2}=484$ and $22^{3}=10\,648$, respectively. When K=4, the size of the vocabulary is $22^{4}=234\,256$, yielding an exponential increase in model complexity. Therefore, this study only evaluated the performance of K=1, 2 and 3. During the K-value analysis, we kept all other parameters fixed, including the epoch number, learning rate, batch size, etc. We adjusted the K-value to perform experiments and determined the optimal K-value according to DTI prediction performance under cross-validation. As shown in Fig. 6, the model with K=2 performed best on ACC and AUC metrics. Therefore, 2-mer sequences are selected to represent proteins.

#### Over-smoothing analysis

**Figure 7:**
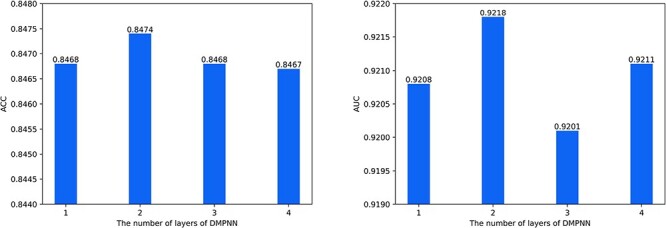
The impact of the number of layers in the D-MPNN module on ACC and AUC.

During the training process, increasing the number of layers of D-MPNN components in the HMSA-DTI causes the hidden states to converge to similar values, producing over-smoothing and thereby decreasing the feature representation ability [43]. Therefore, we performed a range of experiments to assess the influence of the number of layers of the D-MPNN component on the prediction performance of our HMSA-DTI model. During the over-smoothing analysis, the optimal K-value was selected and all the remaining parameters were fixed except for the number of D-MPNN layers. We adjusted the number of D-MPNN layers to perform experiments and selected the optimal number of layers based on DTI prediction performance under cross-validation. As shown in Fig. 7, the HMSA-DTI with D-MPNN of two layers performed best in terms of ACC and AUC metrics. Therefore, we used D-MPNN with two layers in all experiments.

### Case study

In this section, we randomly select a drug and a protein as test candidates. These selected test candidates were then removed from the DrugBank dataset, and the rest were used to construct the training dataset. This validation approach allows us to assess the robustness and generalizability of the HMSA-DTI model and provides insights into its performance in real-world scenarios. In this specific experiment, we randomly chose the drug Estradiol acetate (DrugBankID: DB13952) and the target protein gamma-aminobutyric acid receptor subunit rho-3 (UniprotID: A8MPY1) as the test candidates. We selected 10 proteins from the DrugBank dataset that interact with Estradiol acetate and used them to create 10 positive samples. For the negative samples, we generated 4244 negative samples by combining Estradiol acetate with each of the remaining 4244 proteins in the dataset. The 10 positive samples and 4244 negative samples comprise the testing dataset. We then ranked the candidate target proteins by the predicted interaction scores and listed the top 20 target proteins for evaluation in Table 8. It is demonstrated that the HMSA-DTI successfully predicted all positive samples, five of which ranked among the top 20. Similarly, for the target protein gamma-aminobutyric acid receptor subunit rho-3, we followed the same way to create the test dataset, which consists of 15 positive samples and 6630 negative samples. As shown in Table 9, the HMSA-DTI also accurately predicted all positive samples, three of which are among the top 20. These results show the effectiveness of HMSA-DTI in predicting DTI while showing its robustness and generalizability

**Table 8:** The predicted candidate targets for Estradiol acetate

Rank	Tagget name	UniprotID	Label
**1**	**Estrogen receptor**	**P03372**	**Positive**
**2**	**17-beta-hydroxysteroid dehydrogenase type 2**	**P37059**	**Positive**
3	Mineralocorticoid receptor	P08235	Negative
4	Progesterone receptor	P06401	Negative
5	Glutathione S-transferase P	P09211	Negative
**6**	**Neuronal acetylcholine receptor subunit alpha-4**	**P43681**	**Positive**
**7**	**Estrogen receptor beta**	**Q92731**	**Positive**
8	Vitamin D3 receptor	P11473	Negative
9	Tyrosine-protein phosphatase non-receptor type 1	P18031	Negative
10	Retinoic acid receptor RXR-alpha	P19793	Negative
**11**	**Nuclear receptor coactivator 2**	**Q15596**	**Positive**
12	Sex hormone-binding globulin	P04278	Negative
13	Peroxisome proliferator-activated receptor gamma	P37231	Negative
14	Mu-type opioid receptor	P35372	Negative
15	Neuronal acetylcholine receptor subunit alpha-2	Q15822	Negative
16	Kappa-type opioid receptor	P41145	Negative
17	Gag-Pol polyprotein	P04585	Negative
18	Gag-Pol polyprotein	P03369	Negative
19	Nuclear receptor coactivator 1	Q15788	Negative
20	Peroxisome proliferator-activated receptor alpha	Q07869	Negative

**Table 9:** The predicted candidate drugs for gamma-aminobutyric acid receptor subunit rho-3

Rank	Drug name	DrugBankID	Label
**1**	**Triazolam**	**DB00897**	**Positive**
2	Arsenic trioxide	DB01169	Negative
3	Econazole	DB01127	Negative
4	Acamprosate	DB00659	Negative
5	Miconazole acid	DB03814	Negative
6	Neuronal acetylcholine receptor	DB01110	Negative
7	Enflurane	DB00228	Negative
8	Tetrafluoroaluminate Ion	DB04444	Negative
9	Halothane	DB01159	Negative
10	(5S)-3-ANILINO-5-(2,4-DIFLUOROPHENYL)-5-METHYL-1,3-OXAZOLIDINE 2,4-DIONE	DB07763	Negative
11	Trapidil	DB01189	Negative
12	Desflurane	DB01189	Negative
13	Isoflurane	DB00753	Negative
14	Ketazolam	DB01587	Negative
15	Mitotane	DB00648	Negative
16	Tioconazole	DB01007	Negative
17	N,N-Bis(4-Chlorobenzyl)-1h-1,2,3,4-Tetraazol-5-Amine	DB04037	Negative
**18**	**Diazepam**	**DB00829**	**Positive**
**19**	**Fludiazepam**	**DB01567**	**Positive**
20	mu4-sulfido-quadro-tetracopper	DB03151	Negative

## Conclusion

In this study, we present a hierarchical multimodal self-attention-based GNN for DTI prediction, namely HMSA-DTI. HMSA-DTI utilizes multimodal data to improve its feature representation ability and employs a hierarchical multimodal self-attention feature fusion approach to fuse node-level features of molecular graphs with SMILES sequence features, yielding more robust drug features. In addition, a protein sequence representation is combined with a protein 2-mer sequence representation, and their features are fused by hierarchical multimodal self-attention to enhance protein representation ability. We validated our HMSA-DTI model on five benchmark datasets: DrugBank, Human, C. elegans, BioSNAP and Davis. It is shown that our HMSA-DTI model outperforms the baseline models, demonstrating its strong competitiveness and effectiveness.

**Key Points**

- We took multimodal data such as SMILES, drug molecular graphs, protein sequences and 2-mer sequences as inputs. Additionally, the readout phase is removed from D-MPNN to obtain node-level features instead of graph-level features, beneficial to the following multimodal feature fusion.
- Hierarchical multimodal self-attention was proposed to improve the discriminability and robustness of features by computing both intra- and inter-modal interactions.
- Five benchmark datasets were used to validate our proposed HMSA-DTI. It is demonstrated that compared with other baseline models, our proposed HMSA-DTI exhibits superior performance on multiple metrics, and has strong competitiveness.

## Data Availability

Codes and datasets are available at https://github.com/tangjlh/HMSA-DTI.

## Author Biographies

**Jilong Bian** is an associate professor at the College of Computer and Control Engineering, Northeast Forestry University. His research interests include deep learning, bioinformatics and computer vision.

**Hao Lu** is a master student at the College of Computer and Control Engineering, Northeast Forestry University. His research interests include deep learning and bioinformatics.

**Guanghui Dong** is an associate professor at the College of Computer and Control Engineering, Northeast Forestry University. His research interests include bioinformatics and image processing.

**Guohua Wang** is a professor at the College of Computer and Control Engineering, Northeast Forestry University. He is also a Principal Investigator at the Key Laboratory of Tree Genetics and Breeding, Northeast Forestry University. His research interests are bioinformatics, machine learning and algorithms.

## References

[1] Bagherian M , Sabeti E, Wang K. et al. Machine learning approaches and databases for prediction of drug-target interaction: a survey paper. Brief Bioinform 2021;22:247–69. https://doi.org/10.1093/bib/bbz157 31950972 10.1093/bib/bbz157 PMC7820849

[2] Li Y , Qiao G, Wang K. et al. Drug-target interaction predication via multi-channel graph neural networks. Brief Bioinform 2022;23. https://doi.org/10.1093/bib/bbab346 10.1093/bib/bbab346 34661237

[3] Cao D-S , Zhang L-X, Tan G-S. et al. Computational prediction of drug-target interactions using chemical, biological, and network features. Molecular Informatics 2014;33:669–81. https://doi.org/10.1002/minf.201400009 27485302 10.1002/minf.201400009

[4] Shi H , Liu S, Chen J. et al. Predicting drug-target interactions using lasso with random forest based on evolutionary information and chemical structure. Genomics 2019;111:1839–52. https://doi.org/10.1016/j.ygeno.2018.12.007 30550813 10.1016/j.ygeno.2018.12.007

[5] Ahn S , Lee SE, Kim M-h. Random-forest model for drug-target interaction prediction via kullback-leibler divergence. J Chem 2022;14:1–13. https://doi.org/10.1186/s13321-022-00644-1 10.1186/s13321-022-00644-1 PMC9531514 36192818

[6] Jacob L , Vert J-P. Protein-ligand interaction prediction: an improved chemogenomics approach. Bioinformatics 2008;24:2149–56. https://doi.org/10.1093/bioinformatics/btn409 18676415 10.1093/bioinformatics/btn409 PMC2553441

[7] Bleakley K , Yamanishi Y. Supervised prediction of drug-target interactions using bipartite local models. Bioinformatics 2009;25:2397–403. https://doi.org/10.1093/bioinformatics/btp433 19605421 10.1093/bioinformatics/btp433 PMC2735674

[8] Keum J , Nam H. Self-blm: prediction of drug-target interactions via self-training svm. PloS One 2017;12:e0171839. https://doi.org/10.1371/journal.pone.0171839 10.1371/journal.pone.0171839 PMC5305209 28192537

[9] Perlman L , Gottlieb A, Atias N. et al. Combining drug and gene similarity measures for drug-target elucidation. J Comput Biol 2011;18:133–45. https://doi.org/10.1089/cmb.2010.0213 21314453 10.1089/cmb.2010.0213

[10] Kim S , Jin D, Lee H. Predicting drug-target interactions using drug-drug interactions. PloS One 2013;8:e80129. https://doi.org/10.1371/journal.pone.0080129 10.1371/journal.pone.0080129 PMC3836969 24278248

[11] Yang J , He S, Zhang Z. et al. Negstacking: drug-target interaction prediction based on ensemble learning and logistic regression. IEEE/ACM Trans Comput Biol Bioinform 2020;18:2624–34. 10.1109/TCBB.2020.2968025 31985434

[12] Li Y , Qiao G, Gao X. et al. Supervised graph co-contrastive learning for drug-target interaction prediction. Bioinformatics 2022;38:2847–54. https://doi.org/10.1093/bioinformatics/btac164 35561181 10.1093/bioinformatics/btac164

[13] Öztürk H , Özgür A, Ozkirimli E. Deepdta: deep drug-target binding affinity prediction. Bioinformatics 2018;34:i821–9. https://doi.org/10.1093/bioinformatics/bty593 30423097 10.1093/bioinformatics/bty593 PMC6129291

[14] Lee I , Keum J, Nam H. Deepconv-dti: prediction of drug-target interactions via deep learning with convolution on protein sequences. PLoS Comput Biol 2019;15:e1007129. https://doi.org/10.1371/journal.pcbi.1007129 10.1371/journal.pcbi.1007129 PMC6594651 31199797

[15] Abbasi K , Razzaghi P, Poso A. et al. Deepcda: deep cross-domain compound-protein affinity prediction through lstm and convolutional neural networks. Bioinformatics 2020;36:4633–42. https://doi.org/10.1093/bioinformatics/btaa544 32462178 10.1093/bioinformatics/btaa544

[16] Vaswani A , Shazeer N, Parmar N. et al. Attention is all you need. Advances in Neural Information Processing Systems 2017;30:5990–6008.

[17] Chen L , Tan X, Wang D. et al. Transformercpi: improving compound-protein interaction prediction by sequence-based deep learning with self-attention mechanism and label reversal experiments. Bioinformatics 2020;36:4406–14. https://doi.org/10.1093/bioinformatics/btaa524 32428219 10.1093/bioinformatics/btaa524

[18] Tsubaki M , Tomii K, Sese J. Compound-protein interaction prediction with end-to-end learning of neural networks for graphs and sequences. Bioinformatics 2018;35:309–18. https://doi.org/10.1093/bioinformatics/bty535 10.1093/bioinformatics/bty535 29982330

[19] Li F , Zhang Z, Guan J. et al. Effective drug–target interaction prediction with mutual interaction neural network. Bioinformatics 2022;38:3582–9. https://doi.org/10.1093/bioinformatics/btac377 35652721 10.1093/bioinformatics/btac377 PMC9272808

[20] Wang X , Liu D, Zhu J. et al. Csconv2d: a 2-d structural convolution neural network with a channel and spatial attention mechanism for protein-ligand binding affinity prediction. Biomolecules 2021;11:643. https://doi.org/10.3390/biom11050643 33925310 10.3390/biom11050643 PMC8145762

[21] Rifaioglu AS , Nalbat E, Atalay V. et al. Deepscreen: high performance drug–target interaction prediction with convolutional neural networks using 2-d structural compound representations. Chem Sci 2020;11:2531–57. https://doi.org/10.1039/C9SC03414E 33209251 10.1039/c9sc03414e PMC7643205

[22] Torng W , Altman RB. Graph convolutional neural networks for predicting drug-target interactions. J Chem Inf Model 2019;59:4131–49. https://doi.org/10.1021/acs.jcim.9b00628 31580672 10.1021/acs.jcim.9b00628

[23] Bai P , Miljkovi’c F, John B. et al. Interpretable bilinear attention network with domain adaptation improves drug–target prediction. *Nature*. Machine Intelligence 2022;5:126–36. https://doi.org/10.1038/s42256-022-00605-1

[24] Huang Y , Chenzhuang D, Xue Z. et al. What makes multi-modal learning better than single (provably). In Advances in Neural Information Processing Systems 2021;34:10944–56.

[25] Yifan W , Gao M, Zeng M. et al. Bridgedpi: a novel graph neural network for predicting drug-protein interactions. Bioinformatics 2022;38:2571–8. 35274672 10.1093/bioinformatics/btac155

[26] Wang H , Zhu H, Li W. et al. Predicting compound-protein interaction by deepening the systemic background via molecular network feature embedding. In *2022 IEEE International Conference on Bioinformatics and Biomedicine (BIBM)*, p. 346–53, 2022.

[27] Hua Y , Song X, Feng Z. et al. Cpinformer for efficient and robust compound-protein interaction prediction. IEEE/ACM Trans Comput Biol Bioinform 2022;20:1–96. https://doi.org/10.1109/TCBB.2022.3144008 10.1109/TCBB.2022.3144008 35044921

[28] Zhou H , Zhang S, Peng J. et al. Informer: beyond efficient transformer for long sequence time-series forecasting. In Proceedings of the AAAI Conference on Artificial Intelligence, Vol. 35, p. 11106–15, 2021, https://doi.org/10.1609/aaai.v35i12.17325.

[29] Min X , Zeng W, Chen N. et al. Chromatin accessibility prediction via convolutional long short-term memory networks with k-mer embedding. Bioinformatics 2017;33:i92–101. https://doi.org/10.1093/bioinformatics/btx234 28881969 10.1093/bioinformatics/btx234 PMC5870572

[30] Rao R , Bhattacharya N, Thomas N. et al. Evaluating protein transfer learning with tape. In Advances in Neural Information Processing Systems 2019;32:9686–98. PMC7774645 33390682

[31] Zhao Q , Zhao H, Zheng K. et al. Hyperattentiondti: improving drug-protein interaction prediction by sequence-based deep learning with attention mechanism. Bioinformatics 2022;38:655–62. https://doi.org/10.1093/bioinformatics/btab715 34664614 10.1093/bioinformatics/btab715

[32] Yang K , Swanson K, Jin W. et al. Analyzing learned molecular representations for property prediction. J Chem Inf Model 2019;59:3370–88. https://doi.org/10.1021/acs.jcim.9b00237 31361484 10.1021/acs.jcim.9b00237 PMC6727618

[33] Yang Z , Zhong W, Lv Q. et al. Learning size-adaptive molecular substructures for explainable drug–drug interaction prediction by substructure-aware graph neural network. Chem Sci 2022;13:8693–703. https://doi.org/10.1039/D2SC02023H 35974769 10.1039/d2sc02023h PMC9337739

[34] Stahlschmidt S , Ulfenborg B, Synnergren J. Multimodal deep learning for biomedical data fusion: a review. Brief Bioinform 2022;23:bbab569. 35089332 10.1093/bib/bbab569 PMC8921642

[35] Liu H , Sun J, Guan J. et al. Improving compound–protein interaction prediction by building up highly credible negative samples. Bioinformatics 2015;31:i221–9. https://doi.org/10.1093/bioinformatics/btv256 26072486 10.1093/bioinformatics/btv256 PMC4765858

[36] Zitnik M , Sosič, Leskovec J. Biosnap datasets: Stanford biomedical network dataset collection, 2018.

[37] Davis M , Hunt J, Herrgård S. et al. Comprehensive analysis of kinase inhibitor selectivity. Nat Biotechnol 2011;29:1046–51. https://doi.org/10.1038/nbt.1990 22037378 10.1038/nbt.1990

[38] Zhao Q , Duan G, Zhao H. et al. Gifdti: prediction of drug-target interactions based on global molecular and intermolecular interaction representation learning. IEEE/ACM Trans Comput Biol Bioinform 2023;20:1943–52. https://doi.org/10.1109/TCBB.2022.3225423 36445997 10.1109/TCBB.2022.3225423

[39] Huang L , Lin J, Liu R. et al. Coadti: multi-modal co-attention based framework for drug-target interaction annotation. Brief Bioinform 2022;23:bbac446. 36274236 10.1093/bib/bbac446

[40] Cheng Z , Yan C, Fang-Xiang W. et al. Drug-target interaction prediction using multi-head self-attention and graph attention network. IEEE/ACM Trans Comput Biol Bioinform 2022;19:2208–18. https://doi.org/10.1109/TCBB.2021.3077905 33956632 10.1109/TCBB.2021.3077905

[41] Wang X , Girshick R, Gupta A. et al. Non-local neural networks. In 2018 IEEE/CVF Conference on Computer Vision and Pattern Recognition, p. 7794–803, 2018.

[42] Yaohua P , Yijia Z, Jing Z. et al. Csdti: an interpretable cross-attention network with gnn-based drug molecule aggregation for drug-target interaction prediction. Applied Intelligence 2023;53:27177–90.

[43] Chen D , Lin Y, Li W. et al. Measuring and relieving the over-smoothing problem for graph neural networks from the topological view. In Proceedings of the AAAI Conference on Artificial Intelligence, Vol. 34, p. 3438–45, 2020, https://doi.org/10.1609/aaai.v34i04.5747.

